# Effect of joint line orientation parameters on initial bone resection in mechanically aligned total knee arthroplasty: a retrospective clinicoradiological correlation study

**DOI:** 10.1186/s12891-023-06299-9

**Published:** 2023-03-24

**Authors:** Liang Wen, Yang Yu, Desi Ma, Zhiwei Wang

**Affiliations:** Department of Orthopedics, Beijing Chaoyang Hospital, Capital Medical University, 100020 Beijing, China

**Keywords:** Joint line, Orientation parameters, Bone resection, Osteoarthritis, Total knee arthroplasty

## Abstract

**Background:**

Discrepancies in bone resection between the medial and lateral compartments are very common in total knee arthroplasty (TKA) when mechanical alignment (MA) is used. The purpose of this study was to explore whether and how joint line orientation affects the initial bone resection in mechanically aligned TKA.

**Methods:**

A total of 194 patients (225 knees) diagnosed with osteoarthritis (OA) were included. Virtual bone resection was conducted in the coronal view using full-length weight-bearing radiographs according to the technical requirements of MA, and the reliability of the virtual resection was verified via intraoperative caliper measurements. Correlation and regression analyses were conducted between the initial bone resection within the extension gap (EG) and various parameters, including the hip-knee-ankle (HKA) angle, mechanical lateral distal femoral angle (mLDFA), joint line congruence angle (JLCA), and medial proximal tibial angle (MPTA). Moreover, the correlation between intraoperative bone resection adjustments and joint line orientation parameters was also investigated.

**Results:**

All knees in the current case series were artificially divided into 4 subgroups: subgroup 1, containing 148 varus knees (65.8%) with valgus femurs; subgroup 2, containing 48 varus knees (21.3%) with varus femurs; subgroup 3, containing 17 valgus knees (7.6%) with varus tibias; and subgroup 4, containing 12 valgus knees (5.3%) with valgus tibias. In subgroup 1, the mLDFA and MPTA were positively correlated with the initial bone resection with regression coefficients of 0.670 and 0.089, respectively. Moreover, in all varus knees, intraoperative bone resection adjustments were negatively correlated with mLDFA and MPTA, with categorical regression coefficients of -0.426 and − 0.230, respectively.

**Conclusion:**

When MA-TKAs are performed in varus knees with valgus femurs, the initial bone resection within the EG is mainly positively correlated with mLDFA, while the intraoperative bone resection adjustment is significantly correlated with mLDFA and MPTA in all varus knees.

## Background

In mechanically aligned total knee arthroplasty (TKA), the thickness of bone resected from the medial and lateral condyle of the distal femur has been reported to be significantly different [1, 2]. Similarly, the thickness of bone resected from the medial and lateral tibial plateau differed as well [3]. This is because the mechanical alignment (MA) technique necessitates the implantation of femoral and tibial components perpendicular to the mechanical axis of the femur and tibia, respectively, and does not take the variations of the joint line orientation into full consideration [4, 5].

In the more recent coronal alignment classifications of the knee that integrate the joint line orientation parameters, if 3° is used as the range of the knee phenotype, then the knee phenotype that fully meets MA is only 5.6% and 3.6% in the male and female populations, respectively [6]. In contrast, if 4° is used as the range, then the knee phenotype that meets MA is about 15% [7]. These results suggest that MA significantly alters the native joint line orientation in most people.

Such changes in the native joint line orientation inevitably lead to discrepancies in medial-lateral compartment resection of the knee and consequently to imbalances in the extension gap (EG), especially when conventional instruments are used to perform MA-TKA. So, how does the native joint line orientation affect the initial bone resection of MA-TKA in the coronal plane? Is it possible to predict the amount of intraoperative bone resection by measuring parameters related to the knee joint line orientation preoperatively? To our knowledge, no such studies have been reported. Before specifying this question, the parameters representing the knee joint line orientation should be defined first. The mechanical lateral distal femoral angle (mLDFA), joint line congruence angle (JLCA) and medial proximal tibial angle (MPTA) were chosen as parameters describing the knee joint line orientation as per the approach described by Paley et al [8]. While the hip-knee-ankle (HKA) angle was included in the analysis because it is an important parameter describing the alignment of the lower extremity. The hypothesis of this study is that these joint line orientation parameters would impact the initial bone resection of MA-TKA on the coronal plane and possibly lead to intraoperative bone resection adjustments that would have to be implemented.

## Methods

The current study is a correlation analysis using retrospective radiological measurements and the virtual bone resection used to create the EG. Joint line orientation parameters were measured on an anteroposterior full-length weight-bearing roentgenogram. Bone resection simulation was performed to evaluate the gross bone loss. The relationship between various joint line orientation parameters and the supposed bone resection was investigated to judge the impact of joint line orientation parameters on initial bone resection or even intraoperative bone recuts in TKA.

### Patients

**Fig. 1:**
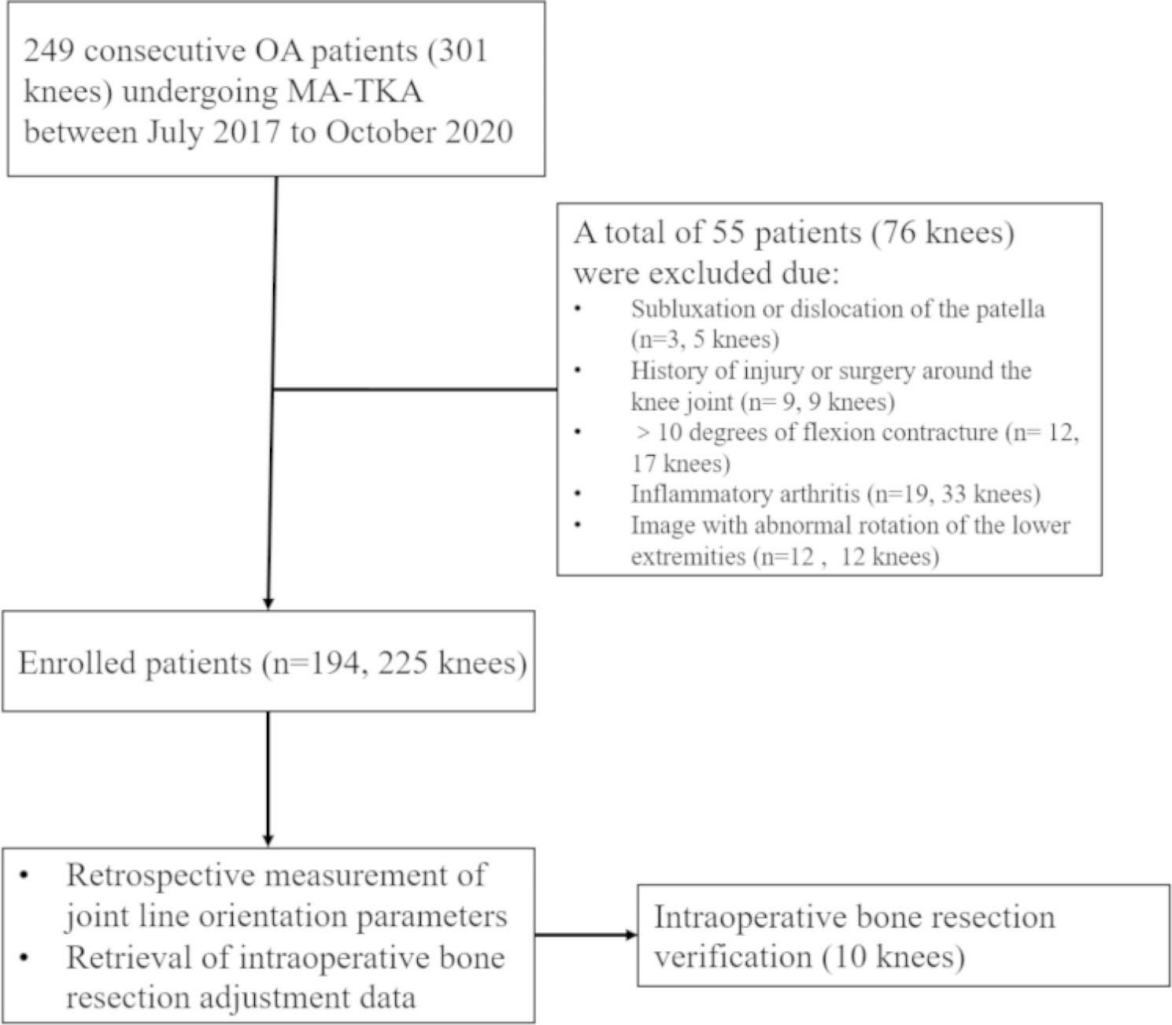
Flow chart of patient selection for this retrospective cohort study

To ensure the accuracy of the radiological measurements, the inclusion criteria for this study were Kellgren-Lawrence grade III-IV knee OA and full-length weight-bearing radiographs of both lower extremities in which the projection of the patella was centered between the femoral condyles. The exclusion criteria included fixed subluxation or dislocation of the patella, congenital lower extremity deformity, history of injury or surgery around the knee joint, significant corrosion of subchondral bone or significant deformity compared with the contralateral knee, > 10 degrees of flexion contracture, inflammatory arthritis, and abnormal rotation (patellar edge beyond the contour of the femoral condyle) of the lower extremities due to technical aspects of imaging. A total of 194 consecutive patients diagnosed with OA undergoing primary TKA (225 knees) at our institute between July 2017 and October 2020 were included in the current study (Fig. 1, patient demographics are shown in Table 1).

### Radiology conditions and setup

Anteroposterior full-length weight-bearing radiographs were obtained using a Definium 8000 digital radiographic system (GE, MA, USA) with an exposure distance of 1.8 m, voltage of 80 kV, and current of 400 mA in auto exposure mode. Patients were usually instructed to stand with the feet together and the patellas facing forward. In the case of tibial torsion, the feet were rotated to orientate the patella forward. The hip, knee and ankle joints were exposed separately, and full-leg weight-bearing radiographs were automatically spliced and synthesized by the system.

### Designation of anatomical landmarks and measurement of joint line parameters

A picture archiving and communication system (PACS) was used to generate radiological measurements in this study. The designation of anatomical landmarks and various joint orientation angles was performed according to the method described by Paley et al. [8]. The center of the femoral head was identified using the Mose circles approach. The center of the distal femur, tibial plateau and ankle joint were defined using the top of the femoral notch, the center of the tibial spine and the mid-width of the talus, respectively. The mechanical axes of the femur and tibia were subsequently determined by connecting the above anatomical landmarks. The distal femoral joint line was designated as the line tangential to the medial and lateral femoral condyles, and the proximal tibial joint line was similarly defined as the line tangential to the concave aspect of the subchondral line of the tibial plateau.

The HKA angle was defined as the angle between the femoral and tibial mechanical axes. A negative HKA angle indicated a varus knee, and a positive angle indicated a valgus knee. The mLDFA was defined as the lateral angle formed by the femoral mechanical axis and the distal femoral joint line. The JLCA was defined as the angle formed by the distal femoral joint line and the proximal tibial joint line. JLCAs with medial convergence were negative values, and JLCAs with lateral convergence were positive values. The MPTA was defined as the medial angle between the mechanical tibial axis and the proximal tibial joint line.

### Measurement of virtual bone resection thickness

**Fig. 2:**
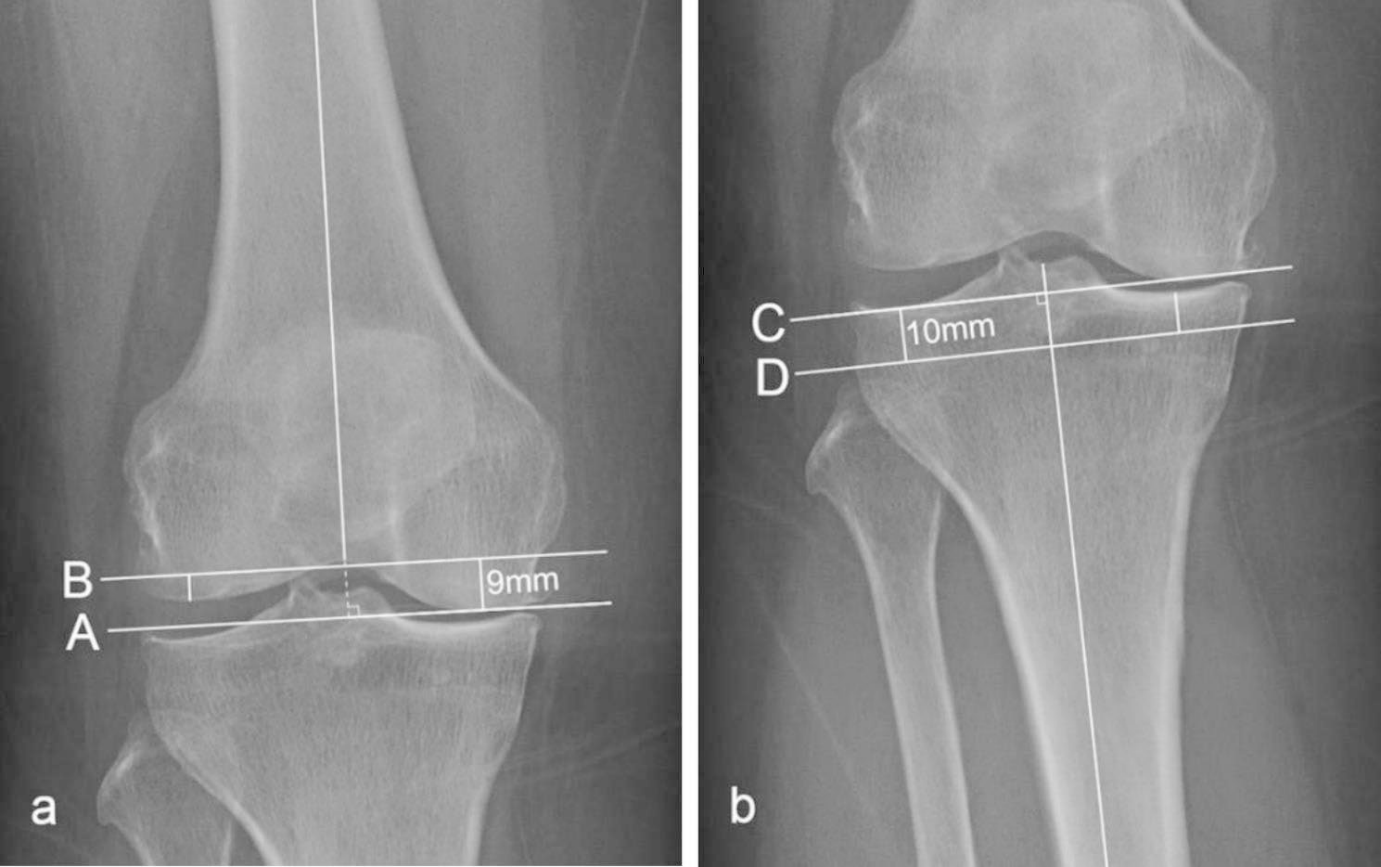
Partial schematics of virtual bone resection on full-length weight-bearing radiographs. The thicknesses of bone resected from the femoral condyles (a) and tibial plateau (b) are shown. Line A is tangential to the most distal portion of the femur and perpendicular to the femoral mechanical axis. Line B is parallel to Line A and 9 mm away from Line A. Line C is tangential to the most proximal articular surface and perpendicular to the tibial mechanical axis. Line D is parallel to Line C and 10 mm below Line C

Virtual bone resection was performed using the PACS as per the mechanical alignment (MA) technique. On the femoral side, a line (Line A) that was both tangential to the most distal point of the femur (either the medial or lateral condyle) and perpendicular to the femoral mechanical axis was drawn. Then, another line (Line B) parallel to Line A and 9 mm proximal to Line A was drawn. Finally, the distances from Line B to the most distal part of the medial and lateral femoral condyles were measured and recorded as the medial and lateral femoral resection thicknesses, respectively (Fig. 2a). On the tibial side, a line (Line C) that was both tangential to the most prominent articular surface (either the medial or lateral plateau) and perpendicular to the tibial mechanical axis was drawn. Similarly, another line (Line D) parallel to Line C and 10 mm distal to Line C was set. The distances from Line D to the two points on the medial and lateral concave aspects of the tibial plateau were measured as the medial and lateral tibial resection thicknesses, respectively (Fig. 2b). The magnification of the radiographs was 1.05. All imaging measurements were performed by two independent physicians (YY, DM). The inter-observer intraclass correlation coefficients (ICC) of the HKA angle, mLDFA, JLCA and MPTA were 0.894 (95% CI: 0.865 ~ 0.918), 0.914 (95% CI: 0.890 ~ 0.933), 0.972 (95% CI: 0.963 ~ 0.978), and 0.537 (95% CI: 0.437 ~ 0.624), respectively. The mean values were utilized in the subsequent statistical analysis.

### Calculation of initial bone resection thickness

The medial resection thickness was calculated as the sum of the medial femoral resection thickness and the medial tibial resection thickness, with the same algorithm for the lateral resection thickness. Regardless of the medial or lateral resection thickness, only the larger value (hereafter referred to as the “max. resection” thickness) was used in the following calculations. The rationale for this algorithm is that in clinical scenarios, to ensure postoperative lower extremity alignment, the distal femoral and proximal tibial resections must be perpendicular to their individual mechanical axes. If the EG is too tight because of less bone resection on one side, it is usually necessary to remove the osteophyte or release the ligament rather than perform additional parallel resection to achieve EG balance.

### Surgical procedure and intraoperative verification

Operations were performed by two senior attending surgeons (ZW, LW). Gemini MK-II prostheses (Link, Hamburger, Germany) were used in this study, of which 215 were the cruciate-retaining type and 10 were the posterior stabilized type. Conventional instruments were used. Distal femoral resection was conducted perpendicular to the femoral mechanical axis [9]. After the valgus resection angle was set accordingly, a bone resection thickness of 9 mm was determined based on the most prominent femoral condyle. Tibial resection was conducted perpendicular to the tibial mechanical axis, and the resection level was estimated using the stylus as 10 mm from the central point of the lateral plateau or the less worn side. The EG was subsequently preliminarily estimated. Measured resection techniques were used in the current study. When the bone preparation was completed, any necessary ligament release was performed before the components were cemented.

**Fig. 3:**
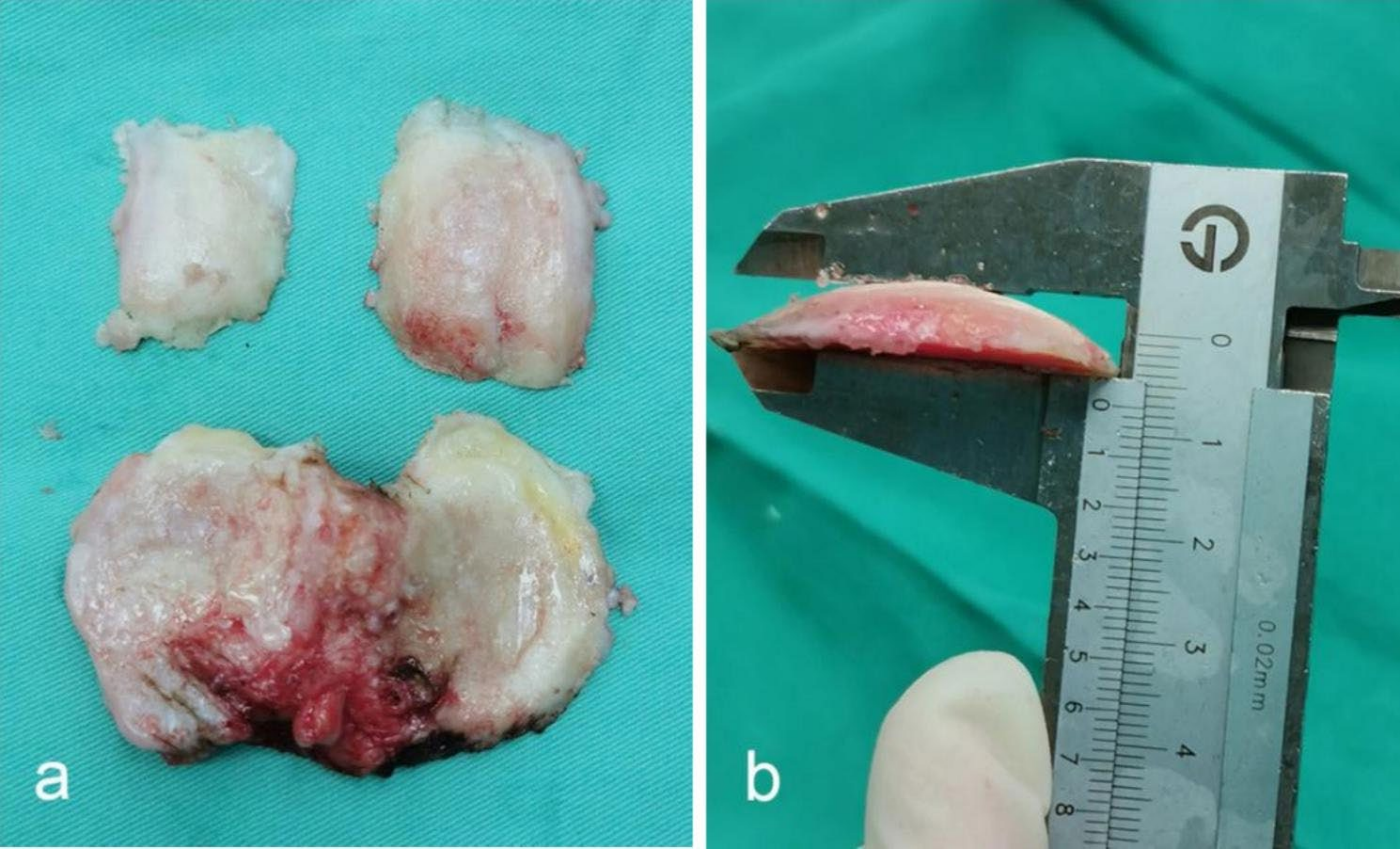
Demonstration of bone pieces resected in TKA (a) and measurement of the resection thickness with a caliper (b)

Intraoperative resection data were randomly measured in 10 patients in this study to verify the accuracy of the bone resection simulation. 4 numerical variables were measured in each case: measures of the medial femoral condyle, lateral femoral condyle, medial tibial plateau and lateral tibial plateau(Fig. 3a, b). A total of 40 paired measurements were tested for intraoperative verification, meaning that the power for distinguishing a difference of 0.5 in the ICC exceeded 90% [10]. Intraoperative measurements were required to compensate for the oscillating saw kerf (1.5 mm), partial wear (1 mm) and complete wear (2 mm) of cartilage, respectively.

### Retrospective collection of bone resection data

The original medical records of all included patients were retrospectively analyzed. All operative records were retrieved to obtain detailed information on intraoperative adjustments to the bone resection thickness, including whether the additional cuts were made to the distal femur or proximal tibia, whether a thicker insert was used, and whether an augment was used or thicker bone cement was applied beneath the femoral component to balance the flexion-extension gap.

### Statistical analysis

Data are described using the mean ± standard deviation or median (interquartile range) *M(Q)* based on the data distribution, which was determined via the Shapiro–Wilk test. Correlation analysis was used to investigate the relationship between the max. resection and various joint line orientation parameters. Linear regression analysis was conducted to identify predictors of the max. resection. Categorical regression with optimal scaling was used to examine the relationship between intraoperative bone resection adjustments and joint line orientation parameters. Since the intraoperative bone cut adjustments were nominal data, data conversion into ordinal categorical data was necessary to increase the power of the test. This data conversion, however, has some drawbacks because it is not possible to distinguish whether the bone recut was from the tibial or femoral side. The ICC is generally considered to indicate poor reliability when below 0.4 and good reliability when above 0.7. All statistical tests were performed using SPSS statistical software (version 22; SPSS, Inc., Chicago, IL, USA). A p value of < 0.05 was considered statistically significant. All plots in this study were generated using R (4.0.0, R Core Team (2020), Vienna, Austria) and the ggplot2 package (3.2.1, Wickham (2020), New York, US).

## Results

**Fig. 4:**
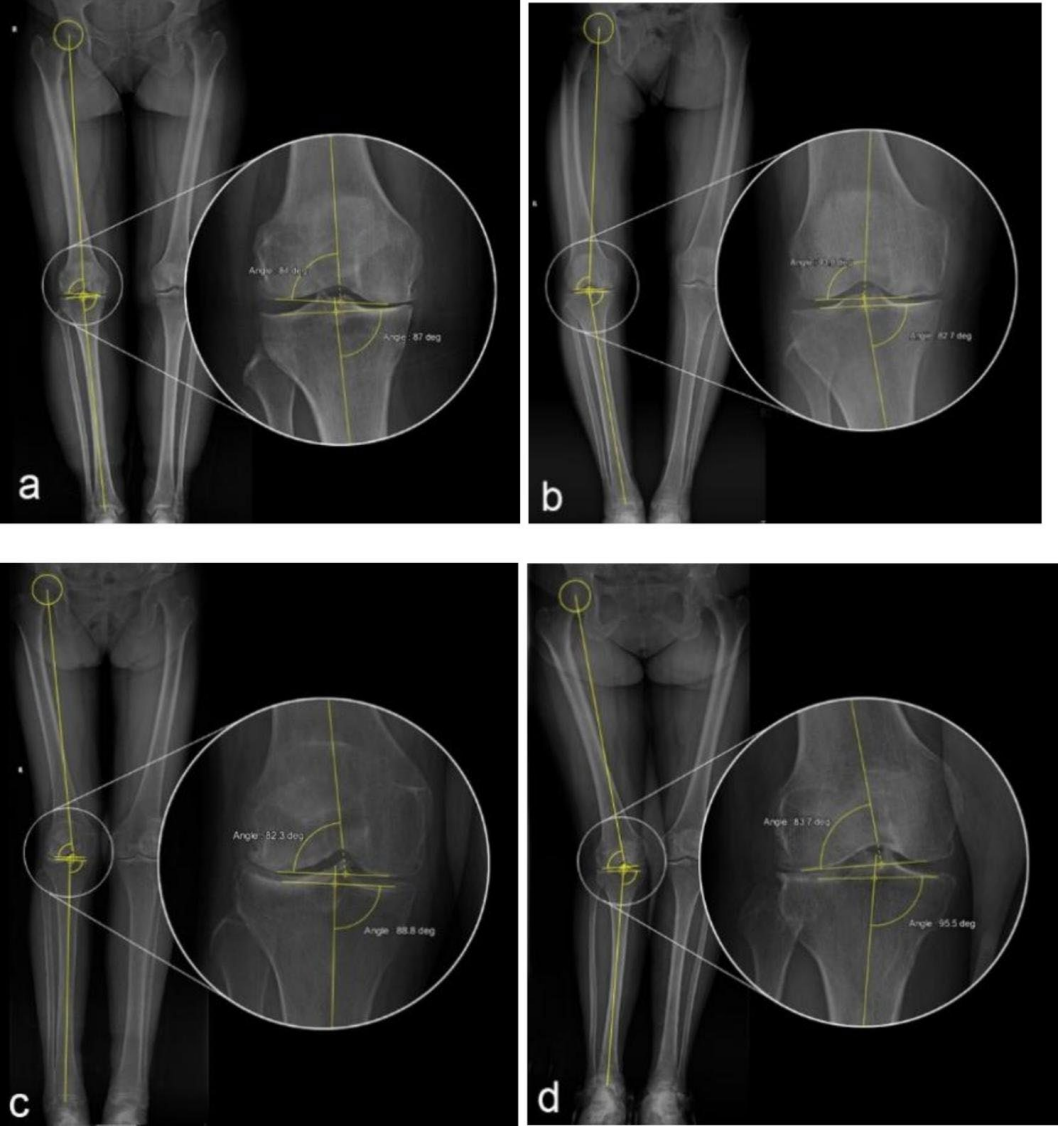
Patients can be divided into 4 subgroups based on limb alignment and joint line orientation, represented by the following: (a) varus knee with mLDFA ≤ 90°; (b) varus knee with mLDFA > 90°; (c) valgus knee with MPTA ≤ 90°; and (d) valgus knee with MPTA > 90°.

Patient demographics are shown in Table 1. Interestingly, among the 225 knees included in this study, all varus knees showed MPTAs less than 90 degrees, while all valgus knees showed mLDFAs less than 90 degrees. Then, the varus knees can be divided into 2 subgroups based on the mLDFA: subgroup 1, varus knees with mLDFA ≤ 90°; and subgroup 2, varus knees with mLDFA > 90°. Similarly, valgus knees can be divided into 2 subgroups based on the MPTA: subgroup 3, valgus knees with MPTA ≤ 90°; and subgroup 4, valgus knees with MPTA > 90° (Fig. 4). The joint line orientation parameters of each group are detailed in Table 2.

**Table 1:** Patient demographics and radiographic joint line parameters

Number of patients	194
Sex (male/female)	56/138
Age (years, *M(Q)*)	69(13)
Number of knees	225
Side (left/right)	109/116
Deformity (varus/valgus)	196/29

**Table 2:** Radiological measurement parameters for each subgroup (mean ± SD or *M(Q)*)

Grouping: n(percentage)	HKA(deg.)	mLDFA(deg.)	JLCA(deg.)	MPTA(deg.)
Overall: 225(100%)	-7.2(5.4)	88.1 ± 2.6	-4.4(3.0)	85.7(3.1)
Subgroup 1: 148(65.8%)	-7.1(16.6)	88(7.0)	-7.9 ± 1.8	85.3 ± 2.1
Subgroup 2: 48(21.3%)	-10.7 ± 3.1	91.3(6.2)	-4.6 ± 1.8	85.3 ± 1.9
Subgroup 3: 17(7.6%)	5.4 ± 1.8	83.9 ± 1.1	2.2 ± 1.1	87.1 ± 1.4
Subgroup 4: 12(5.3%)	9.8 ± 3.6	85.1(2.3)	3.8 ± 1.3	90.7(3.5)

**Fig. 5:**
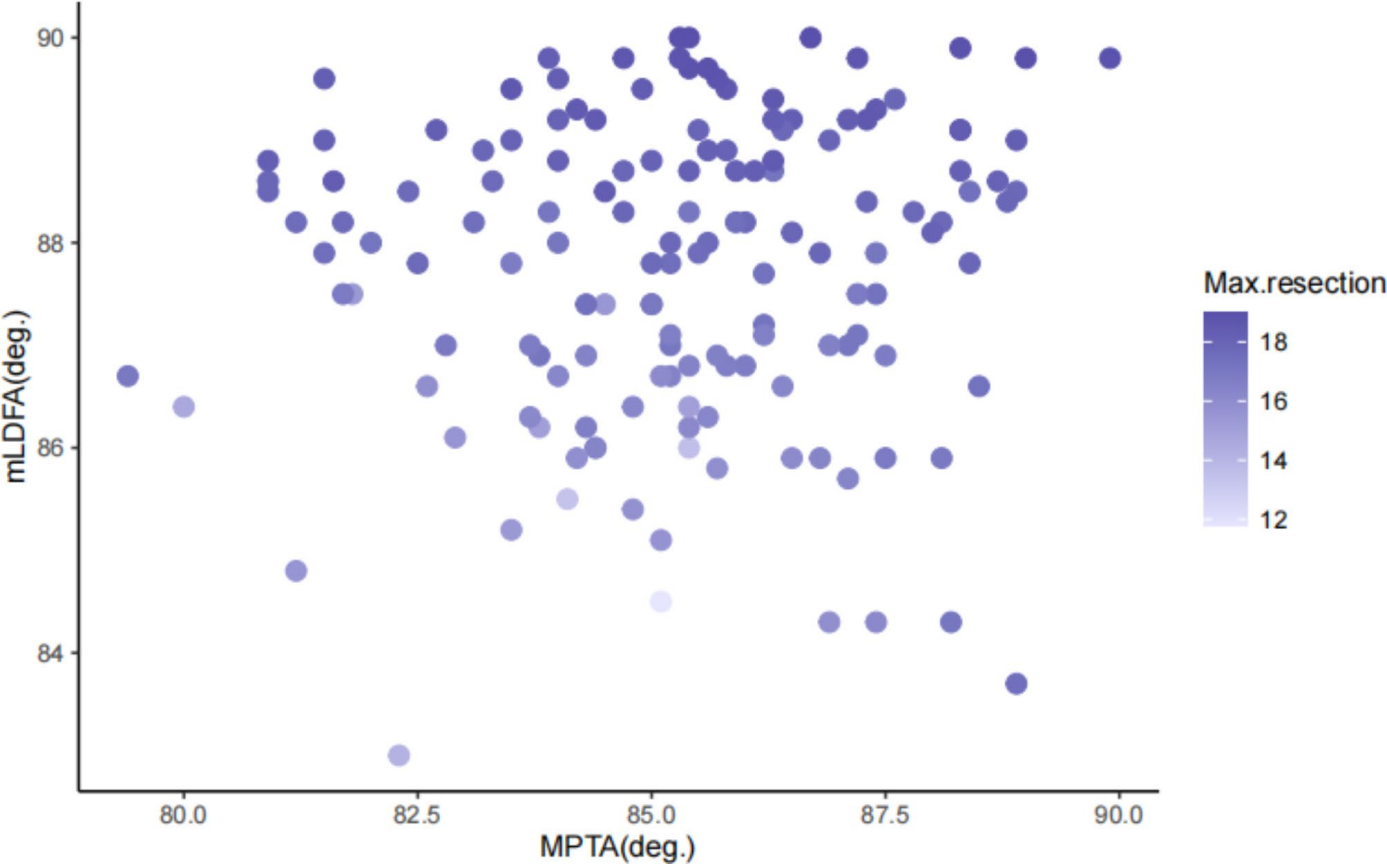
Relationship between the max. resection thickness and joint orientation parameters in varus knees with mLDFA < 90 degrees. Scatter dots weighted by the max. resection thickness are shown. Compared with the light blue dots in the bottom left quadrant, representing fewer bone cuts, the dark blue dots, representing more bone cuts, appear in the upper quadrant rather than the rightmost quadrant. This suggests that the mLDFA has a greater effect on the max. bone resection

**Fig. 6:**
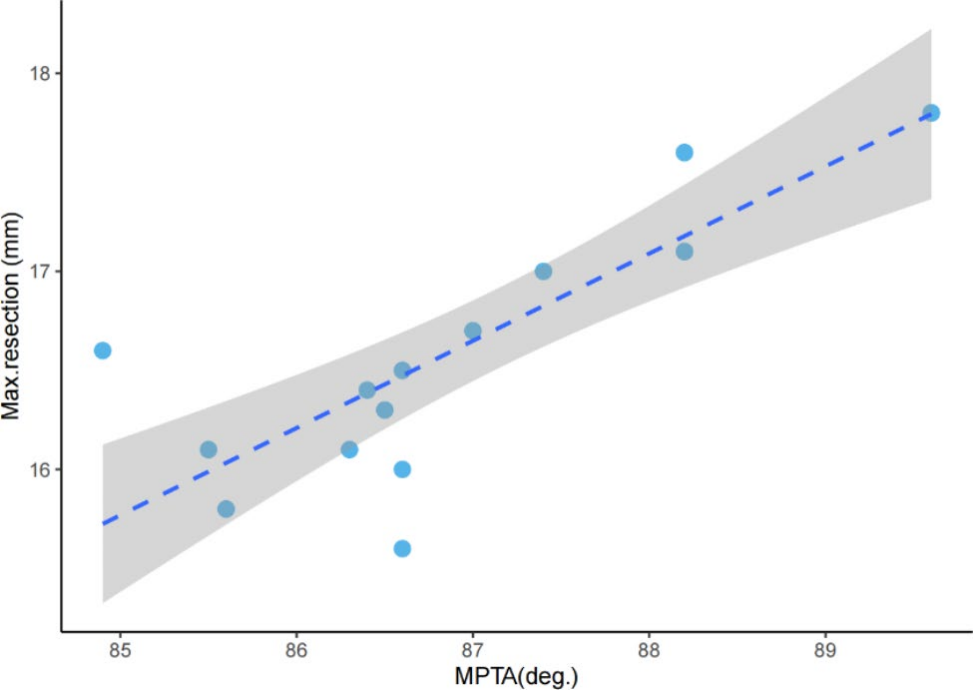
Linear regression plot of the max. resection thickness and MPTA in valgus knees with mLDFA < 90 degrees. The shaded area in the plot indicates the 95% confidence interval

Because the max. resections in subgroups 2 and 4 are constants (the reasons are detailed in the discussion section), only radiological measurements from subgroups 1 and 3 were included in the subsequent analysis. The results of Spearman’s correlation analysis showed that the mLDFA and MPTA were positively correlated with the max. resection thickness in subgroup 1, with correlation coefficients of 0.927 (p < 0.001) and 0.211 (p = 0.01), respectively. Linear regression was subsequently conducted using the mLDFA and MPTA to investigate which parameter had a better predictive capacity. The regression equation was derived as follows: y = 0.670x_1_ + 0.089x_2_-49.163 (R^2^ = 0.730, Durbin-Watson = 2.026), where y represents the max. resection thickness, and x_1_ and x_2_ represent the mLDFA and MPTA, respectively (Fig. 5). On the other hand, correlation analysis showed that only the MPTA had a positive correlation with the max. resection thickness in subgroup 3, with a correlation coefficient of 0.844. The regression equation is y = 0.440x-21.658, R^2^ = 0.712 (Fig. 6).

The ICC of intraoperative verification was 0.984 (95% CI: 0.969 ~ 0.991). Statistical analysis showed the virtual bone resection data in the PACS were highly consistent with the intraoperative measurements.

A retrospective surgical record survey showed that 68.4% of patients did not receive further adjustments (0) following the initial bone resection, while other patients received various intraoperative adjustments, including 2 mm of further tibial resection (t + 2 mm), 2 mm of further distal femoral resection (f + 2 mm), both (+ 4 mm), and a 2-mm-thicker liner (t-2 mm). Very rarely, approximately 2 mm of additional cement was applied between the distal femur and the component to address a relatively wide EG (f-2 mm) (Table 3). No other types of adjustments were found in the review of the surgical records for this case series.

**Table 3:** Intraoperative adjustment of initial bone cut in different subgroups of patients

	f-2 mm	t-2 mm	0	f + 2 mm	t + 2 mm	+ 4 mm
Subgroup 1	0(0%)	9(6.1%)	95(64.2%)	13(8.9%)	22(14.9%)	9(6.1%)
Subgroup 2	2(4.2%)	2(4.2%)	41(85.4%)	1(2.1%)	1(2.1%)	1(2.1%)
Subgroup 3	0(0%)	1(5.9%)	11(64.7%)	3(17.6%)	2(11.8%)	0(0%)
Subgroup 4	2(16.7%)	0(0%)	7(58.3%)	1(8.3%)	2(16.7%)	0(0%)
Total	4(1.8%)	12(5.3%)	154(68.4%)	18(8.0%)	27(12.0%)	10(4.4%)

Note: () is the percentage of patients in each group with this type of bone cut adjustment

**Fig. 7:**
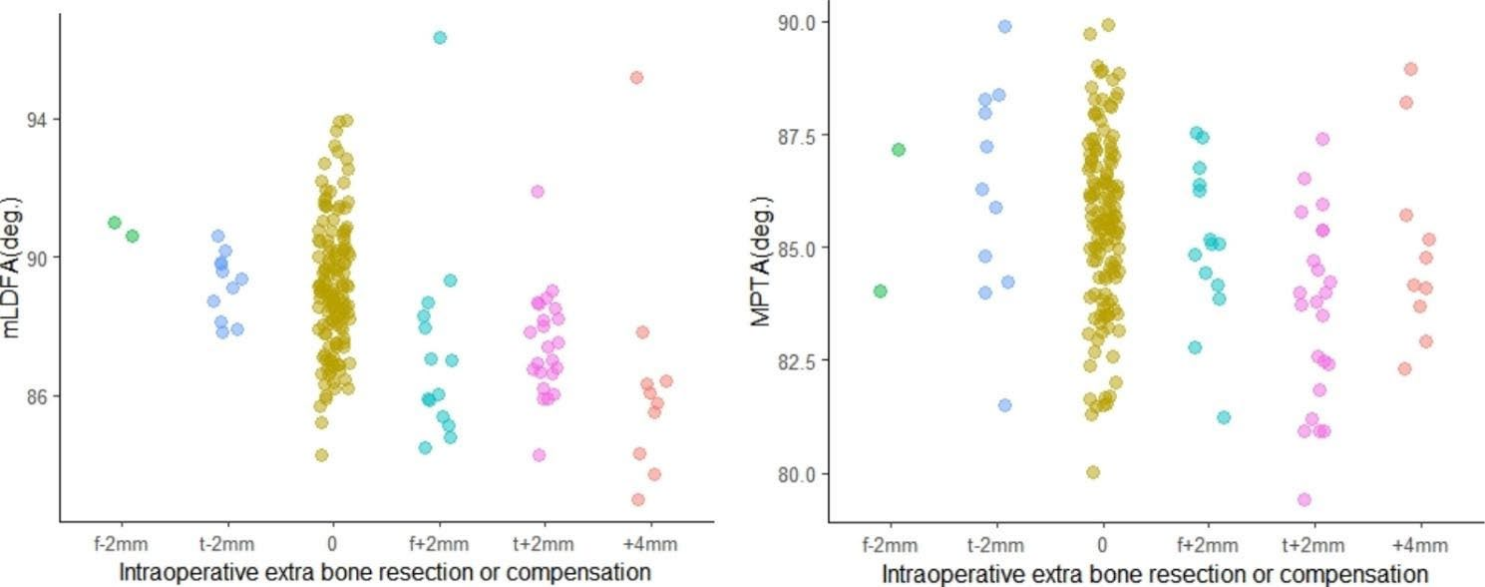
Relationship of intraoperative adjustment of bone resection with mLDFA and MPTA in varus knee Note: 0, no bone resection adjustment; t + 2 mm, 2 mm of additional tibial resection; f + 2 mm, 2 mm of additional distal femoral resection; +4 mm, a total of 4 mm of additional bone resection from either the femoral or tibial side; t-2 mm, application of a 2-mm-thicker liner; f-2 mm, approximately 2 mm thicker cement is applied between the distal femur and the component

Categorical regression with optimal scaling was performed for all subgroups because bone cut adjustments were common. The results showed that all bone cut adjustments that occurred in the varus knees, regardless of the subgroup, showed a negative correlation with mLDFA and MPTA. Furthermore, the contribution of mLDFA to this relationship far exceeded that of MPTA (Table 4). In contrast, all intraoperative bone cut adjustments in the valgus knees did not show any correlation with joint line orientation parameters. To more clearly demonstrate such a relationship in the varus knees, grouped scatter plots were drawn (Fig. 7). As the LDFA and MPTA decrease (i.e., the more oblique the femoral and tibial joint line), the intraoperative bone recut has a tendency to increase.

**Table 4:** Categorical regression performed between intraoperative bone cut adjustments and joint line orientation parameters in this case series

		R^2^	*p*	Predictors	Std. coefficients	Importance*
Varus knees	All varus knees	0.241	0.000	mLDFA	-0.426	0.766
				MPTA	-0.230	0.234
	Subgroup 1	0.300	0.000	mLDFA	-0.484	0.809
				MPTA	-0.222	0.191
	Subgroup 2	0.197	0.007	mLDFA	0.436	0.976
				MPTA	-0.049	0.024
Valgus knees	All valgus knees	0.102	0.247	mLDFA	-0.273	0.824
				MPTA	-0.091	0.176
	Subgroup 3	0.164	0.285	mLDFA	-0.449	1.035
				MPTA	0.160	-0.036
	Subgroup 4	0.201	0.364	mLDFA	-0.459	1.021
				MPTA	0.034	-0.021

Note: *represents the importance percentage of the effect of the independent variables in the model

## Discussion

The main finding of this study was that when TKA was performed with MA using conventional instrumentation on a varus knee with an mLDFA less than 90°, the initial bone resection within the EG was linearly related to the patient’s mLDFA and MPTA, while mLDFA had a more significant impact on the initial bone resection. In addition, intraoperative recut also showed a certain negative correlation with mLDFA and MPTA in the varus knees. The clinical significance is the preoperative acquisition of knee line orientation parameters, such as mLDFA, MPTA, etc., to predict the amount of bone resection within the EG during MA-TKA and to estimate the possibility of intraoperative bone recut.

In the current study, all varus knees were found to have MPTAs less than 90°, and all valgus knees had mLDFAs less than 90°. I.e., when the MA technique is used in TKA for bone preparation, the thickness of lateral tibial plateau resection is always thicker than that of medial resection in varus knees, while the thickness of medial femoral condyle resection is always greater than that of lateral condyle resection in valgus knees. This finding is consistent with those of previously published literatures that the major contributors to valgus and varus knee deformity are valgus of the distal femur [11] and varus of the proximal tibia [2], respectively.

In the present case series, patients in the varus knee group were divided into 2 subgroups based on whether their mLDFA was less than 90°, while those in the valgus knee group were similarly divided with reference to the MPTA. Such grouping is necessary before further correlation analysis. For example, in subgroup 2, the resection thicknesses of the distal femur and tibial plateau were referenced to the lateral condyle and lateral plateau, respectively. the max. resection of subgroup 2 is a constant resection from the lateral compartment. For the same reason, the max. resection of subgroup 4 is also constant.

To the best of our knowledge, no prior studies have focused on the relationship between joint line orientation and the extent of bone resection performed. One study found a significant correlation between the leg axis and the optimal tibial resection thickness, and the optimal resection thickness in valgus knees was significantly less than that in neutral or varus knees; however, no joint line orientation parameters were considered [3]. Inconsistent with the results of the above study, the initial bone resection thickness was not related to the HKA angle in this study. The reason for this difference may be because joint line orientation parameters and simulated resection data for only subgroups 1 and 3 were included in this correlational analysis.

Patients in subgroup 1 had varus knees with valgus femurs and accounted for the majority of all included patients (65.8%). Correlational analysis showed that both the MPTA and mLDFA were positively correlated with the extent of bone resection. The results of the subsequent regression analysis revealed that the mLDFA, rather than the MPTA, had a significant effect on the amount of bone resection. Such results can be interpreted as the majority of patients in the varus knees with an valgus femur have an MPTA much smaller than mLDFA and completely counteract the effect of JLCA. This was indeed the case, as only 20 of 148 patients in subgroup 1 (13.5%) have greater MPTA than mLDFA. For the rest of the patients, max. resection was mainly determined by the bone resection in the lateral compartment, so mLDFA is a better predictor of bone resection in this subgroup.

Another important finding of this study is that intraoperative bone recut is negatively correlated with mLDFA and MPTA in all varus knees, and similarly, still mLDFA has a better predictive role. Although a certain trend of increasing intraoperative recut is found as the mLDFA decreases, However, we are unable to predict a threshold of mLDFA, beyond which an intraoperative recut is likely to be necessary. This is because the data ranges of mLDFA corresponding to the bone cut adjustments in each group overlap significantly (Fig. 7). This suggests that intraoperative bone cut adjustments are influenced by other factors besides joint line parameters, i.e. errors from instruments, individual variability in soft tissue laxity, errors in surgical technique, operator’s preference, etc. No such correlation was found in valgus knees, probably due to the small number of patients.

Our study has several limitations. First, a small number of patients were included in our study. There may be some selection bias, as we excluded some patients due to rotation of the lower extremities in full-length weight-bearing radiographs, and abnormal rotation of the lower extremities results in inaccurate measurement of the MPTA [12]. In addition, because of the limited sample size, it may be that not all patients diagnosed with knee OA can be classified into 4 subgroups. One study reported a valgus knee with an mLDFA as large as 92° [13]. Also because of the small number of cases, no further case stratification was conducted based on the severity of OA in this study, and varying degrees of OA may have an impact on the results of radiological measurements and analysis. Secondly, some special types of extra-articular deformities, such as tibial or femoral shaft bowing with progression of knee OA, were not used as alignment parameters. Although tibial or femoral bowing could dramatically change the joint line orientation, such deformities rarely need correction during primary TKA; thus, our study did not focus on these unusual extra-articular deformities. Thirdly, due to the limitations of retrospective studies, quantitative data of EG balance after initial resection could not be acquired, and intraoperative resection adjustments do not depend exclusively on the amount of initial resection, but also on individual factors such as the amount of osteophytes and the laxity of the soft tissue. Finally, full-length weight-bearing radiographs were used for radiographic measurement rather than 3D CT, and some studies have proven that the latter has higher accuracy [14]. However, the cost effectiveness of radiographs is much greater. The routine determination of some meaningful findings based on preoperative radiographs before TKA could be helpful for guiding clinical practice.

## Conclusion

In MA-TKA, the initial bone resection within the EG is linearly related to the patient’s mLDFA in varus knees with valgus femurs, the intraoperative bone cut adjustment is significantly correlated with mLDFA and MPTA in varus knees as well. This study suggests that attention should be given to this preoperative radiographic variation to avoid multiple intraoperative bone resections or unnecessary bone loss.

## Data Availability

The datasets generated and/or analysed during the current study are not publicly available due to private patient information in the raw data, but are available from the corresponding author on reasonable request.

## Abbreviations

TKA: Total knee arthroplasty
MA: Mechanical alignment
EG: Extension gap
PACS: Picture archiving and communication system
HKA: Hip-knee-ankle
mLDFA: Mechanical lateral distal femoral angle
JLCA: Joint line congruence angle
MPTA: Medial proximal tibial angle

## References

[1] Victor JM, Bassens D, Bellemans J, Gursu S, Dhollander AA, Verdonk PC (2014). Constitutional varus does not affect joint line orientation in the coronal plane. Clin Orthop Relat Res.

[2] Bellemans J, Colyn W, Vandenneucker H, Victor J (2012). The Chitranjan Ranawat award: is neutral mechanical alignment normal for all patients? The concept of constitutional varus. Clin Orthop Relat Res.

[3] Schnurr C, Csécsei G, Nessler J, Eysel P, König DP (2010). How much tibial resection is required in total knee arthroplasty?. Int Orthop.

[4] Rivière C, Iranpour F, Auvinet E, Aframian A, Asare K, Harris S, Cobb J, Parratte S (2017). Mechanical alignment technique for TKA: are there intrinsic technical limitations?. Orthop Traumatology: Surg Res.

[5] Hess S, Moser LB, Amsler F, Behrend H, Hirschmann MT (2019). Highly variable coronal tibial and femoral alignment in osteoarthritic knees: a systematic review. Knee Surg sports Traumatol arthroscopy: official J ESSKA.

[6] Hirschmann MT, Moser LB, Amsler F, Behrend H, Leclerq V, Hess S (2019). Functional knee phenotypes: a novel classification for phenotyping the coronal lower limb alignment based on the native alignment in young non-osteoarthritic patients. Knee Surg sports Traumatol arthroscopy: official J ESSKA.

[7] MacDessi SJ, Griffiths-Jones W, Harris IA, Bellemans J, Chen DB (2021). Coronal plane alignment of the knee (CPAK) classification. The bone & joint journal.

[8] Paley D. Principles of deformity correction. Springer Science & Business Media; 2002.

[9] Tan H, Wang Y, Long T, Nie B, Mao Z, Yue B (2018). How to accurately determine the distal femoral valgus cut angle in the valgus knee arthroplasty. Int Orthop.

[10] Bujang MA, Baharum N. A simplified guide to determination of sample size requirements for estimating the value of intraclass correlation coefficient: a review. Archives of Orofacial Science 2017, 12(1).

[11] Rossi R, Rosso F, Cottino U, Dettoni F, Bonasia DE, Bruzzone M (2014). Total knee arthroplasty in the valgus knee. Int Orthop.

[12] Jamali AA, Meehan JP, Moroski NM, Anderson MJ, Lamba R, Parise C (2017). Do small changes in rotation affect measurements of lower extremity limb alignment?. J Orthop Surg Res.

[13] Eberbach H, Mehl J, Feucht MJ, Bode G, Sudkamp NP, Niemeyer P (2017). Geometry of the Valgus knee: contradicting the Dogma of a femoral-based deformity. Am J Sports Med.

[14] Eckhoff DG, Bach JM, Spitzer VM, Reinig KD, Bagur MM, Baldini TH, Flannery NM (2005). Three-dimensional mechanics, kinematics, and morphology of the knee viewed in virtual reality. J bone joint Surg Am volume.

